# Matisse: a MATLAB-based analysis toolbox for in situ sequencing expression maps

**DOI:** 10.1186/s12859-021-04302-5

**Published:** 2021-07-31

**Authors:** Sergio Marco Salas, Daniel Gyllborg, Christoffer Mattsson Langseth, Mats Nilsson

**Affiliations:** Science for Life Laboratory, Department of Biochemistry and Biophysics, Stockholm University, 171 65, Solna, Sweden

**Keywords:** In situ sequencing, Spatially resolved transcriptomics, Analysis toolbox, Probabilistic cell typing

## Abstract

**Background:**

A range of spatially resolved transcriptomic methods has recently emerged as a way to spatially characterize the molecular and cellular diversity of a tissue. As a consequence, an increasing number of computational techniques are developed to facilitate data analysis. There is also a need for versatile user friendly tools that can be used for a de novo exploration of datasets.

**Results:**

Here we present MATLAB-based Analysis toolbox for in situ sequencing (ISS) expression maps (*Matisse*). We demonstrate *Matisse* by characterizing the 2-dimensional spatial expression of 119 genes profiled in a mouse coronal section, exploring different levels of complexity. Additionally, in a comprehensive analysis, we further analyzed expression maps from a second technology, osmFISH, targeting a similar mouse brain region.

**Conclusion:**

*Matisse* proves to be a valuable tool for initial exploration of in situ* sequencing* datasets*.* The wide set of tools integrated allows for simple analysis, using the position of individual reads, up to more complex clustering and dimensional reduction approaches, taking cellular content into account. The toolbox can be used to analyze one or several samples at a time, even from different spatial technologies, and it includes different segmentation approaches that can be useful in the analysis of spatially resolved transcriptomic datasets.

**Supplementary Information:**

The online version contains supplementary material available at 10.1186/s12859-021-04302-5.

## Background

The emergence of single-cell RNA-sequencing (scRNA-seq) technologies in the last decade have provided the scientific community with high-throughput gene expression of individual cells within a tissue. This has been fundamental for revealing the molecular and cellular complexity of tissues. With these techniques, novel cell types and developmental pathways have been described and have quickly become a standard approach in molecular biology [1]. However, the lack of spatial information in scRNA-seq data has prompted the rapid development of an increasing number of spatially resolved transcriptomic techniques that aim to characterize cells in situ. Thus far, these approaches have aimed to complement single-cell sequencing, but are now starting to be used to answer data driven biological questions. A wide variety of spatially resolved RNA-based techniques have been developed in recent years using both multiplexed targeted approaches as well as unbiased transcriptome wide approaches [2–7]. Due to this diversity, every technique has focused on developing their own analytical tools [8–14] that, despite being powerful in performing in depth analysis of certain datasets, are not flexible enough to perform wide data-driven analysis.

Among image-based approaches, in situ sequencing (ISS) [7] has proved to be a powerful targeted approach for capturing the expression profiles of large tissues with subcellular resolution, and has been applied to study a range of biological questions [15–18]. The second iteration of ISS, Hybridization-based ISS (HybISS) [19] allows for more versatility and multiplexing capacity further increasing the need for user-friendly tools to fit a wider audience. The high-throughput nature of ISS enables the analysis of many samples together, adding another level of complexity in terms of cross-sample variation analysis and comparisons. As a consequence of the variety of biological questions that can be addressed with ISS, diverse analytical tools are required to extract relevant conclusions. Although different specific analytical functionalities have been developed to explore and visualize ISS datasets [20–24], there is a need for a standardized analysis tool that can be used to analyze any kind of ISS dataset. In order to address this need, we here describe the MATLAB-based Analysis Toolbox for ISS Expression maps (*Matisse*). *Matisse* is a user-friendly toolbox designed to facilitate the analysis and interpretation of ISS datasets. It includes a wide number of tools that can be tuned and modified in order to create personalized and versatile analysis pipelines, able to perform simple analysis to more refined approaches.

## Implementation

**Fig. 1:**
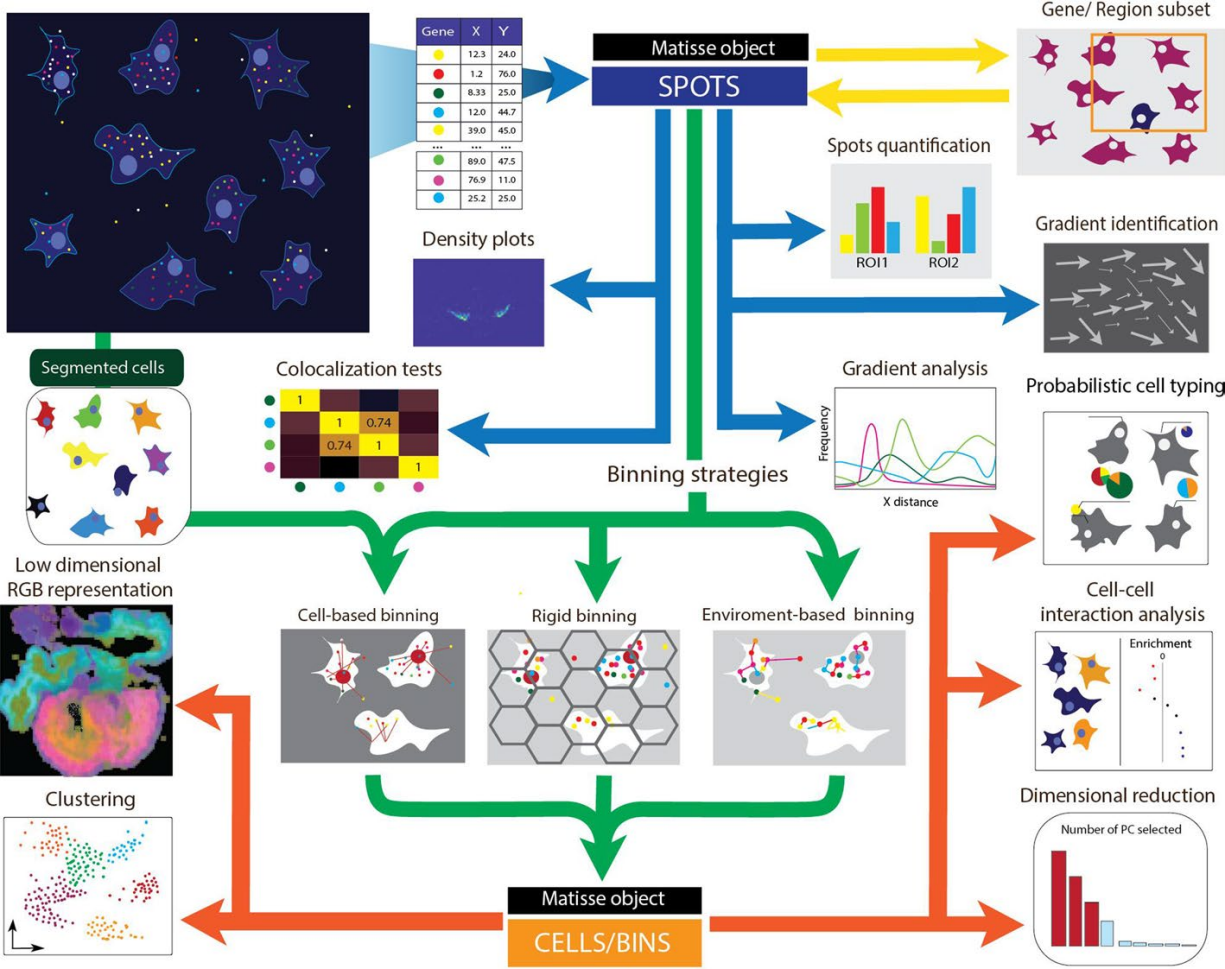
Schematic representation of the main analysis workflow proposed described in MATISSE. The Cartesian coordinates of all reads found in the section analyzed is used to create the initial Matisse object. Several functionalities including KDE plots, colocalization analysis, gene quantification and gradient identification and analysis can be applied using this object as an input. Data can also be segmented, based on the cell boundaries, the location of individual spots or using a grid equally distributed along the section. Its output, stored in a second Matisse object, can be used for cell typing and clustering the data, among others

As with most image-based techniques, preprocessed decoded data comes in the form of a spot table containing 2-dimensional coordinates for each decoded spot, assigned to a specific gene. The input for *Matisse* requires these positions, and additionally, a nuclear staining can be provided to guide cellular segmentation if desired. This input will be formatted into a customized *MatisseMOD* object, which will be used as an input in all the functionalities developed in *Matisse* (Fig. 1). The use of the same object while analyzing the data ensures consistency between the analysis and increases the versatility of the toolbox. A wide number of different analysis can be performed with *Matisse*, including colocalization analysis, Kernel Density Estimation (KDE), and the exploration of gradients for unsegmented datasets, as well as, for example, de novo clustering, dimensional reduction, low dimensional RGB representation, probabilistic cell typing (pciSeq) [20] and gene co-expression for segmented datasets. Several simple segmentation approaches are also implemented to fit the particularities of each dataset. Some of the tools implemented in this toolbox require functions from a previously available repository [https://github.com/Moldia/iss-analysis] which have been incorporated into *Matisse*’s main repository to facilitate its installation.

### Spot quantification

To easily study the amount of reads found in a specific tissue analyzed, the function *PlotTotalCounts* is implemented. Both total counts and relative counts are calculated for all samples/region of interest (ROI) studied by counting the number of reads of a specific gene found in a sample. The count and frequency matrix are represented in bar plots and heat maps, facilitating the interpretation of the results when multiple samples are analyzed at a time. This function was implemented based on the code from a previously available repository [https://github.com/Moldia/iss-analysis].

### Gene density representation

The expression density of a specific gene can be estimated using the function *GeneDensity.* Using the location of the reads detected for a gene, Kernel Density Estimation (KDE) is used to estimate the expression density of a specific gene through the tissue analyzed. The resulting map is represented as a heat map in order to distinguish the different levels of expression found in the sample. This function can be used to analyze several genes at a time. On top of that, the function *OverlayTwoDensities* can be used to overlay the density distribution of different genes. These functions are modifications from previously available utilities [https://github.com/Moldia/iss-analysis].

### Colocalization

The function *Colocalization* is used to study the colocalization of genes within a sample using a neighborhood enrichment test, similar to the algorithm used to identify proximal or interacting cell types used by Dries et al. [25]. For this, a net linking each gene to its closest neighbors is created and the number of connections between genes is calculated. Depending on the characteristics of the dataset, different numbers of neighbors can be specified. To define enrichment or depletion of colocalization, the connections found in the experimental data sets are compared to a set of randomized datasets, containing the same number of reads and positions as the real data but with shuffled read identities. A Z-score will be computed for each pair of genes, computed as follows:

$$Z_{i} = \frac{{x_{i} - \mu }}{\sigma }$$

Therefore, for a given gene *i*, its Z-score will be computed by subtracting the mean number of connections found in the simulated datasets to number of connections found in the real dataset and dividing the resulting value by the standard deviation of the number of connections found in the randomized datasets. Using this method, connections enriched in the real dataset have a positive Z-score, whereas connections depleted in our dataset have a negative Z-score.

### Gradient finder

The function *Find_gradients* is used to identify the main expression gradients found in 2D-expression maps. In order to do this, we calculate the kernel density estimation (KDE) [26] for every gene in the panel assuming a Gaussian kernel and given a specific bandwidth. After that, evenly distributed reference points are positioned through the 2D-dataset. In order to find the higher variations between the different reference points, we calculate both the variation for every spot in the 2 dimensions of the data using:

$$G_{x} = \mathop \sum \limits_{i = 1}^{n} \left| {d_{x - b} - d_{x + b} } \right|$$

In this case, the change in expression in one of the axes (*G*) in a specific spot (*x*) is the summation of the local variation of the *n* genes analyzed on that specific axis. This variation is the absolute value obtained by calculating the difference in density (*d*) of a certain gene in two spots oppositely situated at a specified distance (*b*) from the spot analyzed. This will be computed for both the x axis and the y axis, obtaining two values for each spot analyzed. These two values will be used to generate a quiver or vector plot, showing the direction and intensity of gradients found within the sample.

### Gradient analysis

One dimensional gene expression gradients are analyzed using the function *gradients.* Customizable gradients can be studied using this function. Once the function is called, we define the origin coordinates of the gradient to consider within the image, as well as the size of the region studied. The function calculates the minimum distance of every spot to the origin coordinates, distributing all the reads to a 1-dimensional axis, as it follows:

$$d_{i} = {\text{min}}(\sqrt {(x_{i} - x_{r} )^{2} + (y_{i} - y_{r} )^{2} })$$

Therefore, the distance of every read (*i*) will be the minimum Euclidean distance between the analyzed read and any of the reference spots (*r*). KDE [26] is performed based on the location of every gene’s reads in order to obtain the expression density of genes studied along the gradient. A heat map showing distance of each spot to the origin coordinates and a 1D density plot for the expression density of individual genes is also returned as an output.

### Data segmentation

Different two-dimensional segmentation strategies are used in order to explore the datasets analyzed. *Dapi_segmentation*, adapted from previously available repositories [https://github.com/Moldia/iss-analysis], can be used to segment individual cells using a watershed-based algorithm on a background DAPI image or similar nuclear staining image. A top-hat filter is applied to the image to remove the background and enhance the nuclei, followed by an intensity threshold that will identify the nuclei. Finally, a watershed segmentation is applied, defining individual cells by identifying and expanding the detected nuclear signal. As an output a new mask containing the location and identity of all the cells in the tissue is returned. This mask will consider the parts between cells as unassigned space and reads located within won’t be assigned to a cell. The function *OverlappingBins* is used in order to segment by defining equally distributed bins through the 2-dimensional space analyzed and assigning all reads to the bins they were in. Finally, the function *SpotEnvBin* can be used in order to explore the environment of each read analyzed by defining a bin around each specific read in the dataset summarizing the number of reads found for each gene in within a certain area.

### Principal component analysis and feature selection

Principal components in the dataset are studied using the function *principal_components*, which performs principal component analysis in the desired segmented dataset. Different cell maps representing the scores of the top 20 principal components are created, as well as a heat map representing the loading of gene to those principal components. The variation captured in the main principal components is used to perform dimensionality reduction by selecting those principal components that one finds relevant.

### Low dimensional RGB representation

Low dimensional RGB representation is performed by calling the customized function *LowDimensionalRGB.* Different dimensionality reduction approaches are implemented within the function, including principal component analysis (PCA), t-Distributed Stochastic Neighbor Embedding (t-SNE) and Uniform Manifold Approximation and Projection (UMAP). Both PCA and t-SNE are functions implemented in MATLAB, while UMAP’s implementation was adapted from Meehan et al. [27]. All dimensionality reduction implementations need a cell/gene expression matrix as input, including cells found in all samples, and returns the loadings of the new dimensions regarding each gene, and the score of every cell for the dimensions found. The score of each cell for the three most important dimensions is adjusted to fit into an RGB scale. Individual cells/bins are represented in a spatial map according to their RGB value.

### De novo clustering

De novo clustering requires an initial normalization of the expression matrix obtained after segmenting the dataset. This matrix, containing the number of reads of each gene in each specific cell, is normalized by the total abundance of each gene, removing the effect caused by the high expressers in the clustering. Clustering is performed using the function *Clustering* and requires the specification of the number of clusters desired. Three different clustering algorithms are implemented, including K-means, Hierarchical clustering and DBSCAN [27], returning in all the cases the cluster each cell in the dataset has been assigned to. Further analysis of the clusters found can be done by representing the mean expression of each cluster, using *heatmap_cluster*, or the expression of all the cells assigned to a certain cluster, using *expression_clusters.*

### Probabilistic cell typing

Performing probabilistic cell typing requires the formatting of the reference scRNA-seq data in order to obtain the mean expression of each cell type analyzed for the genes included in the analysis. This data is incorporated in a *matisseMOD* object using the function *load_scRNAseq.* Probabilistic cell typing by in situ sequencing (pciSeq), implemented in the function *pciseq*, requires as an input the *matisseMOD* object summarizing the single cell data and the *matisseMOD* object containing the segmented dataset to cell type. This implementation is adapted from the algorithm described in Qian et al*.* [16] [https://github.com/kdharris101/iss] and returns as an output the cell type map of the sample analyzed, the probabilities of each cell to belong to all cell types, and the gene counts of each cell.

## Results

### Spatial expression analysis

**Fig. 2:**
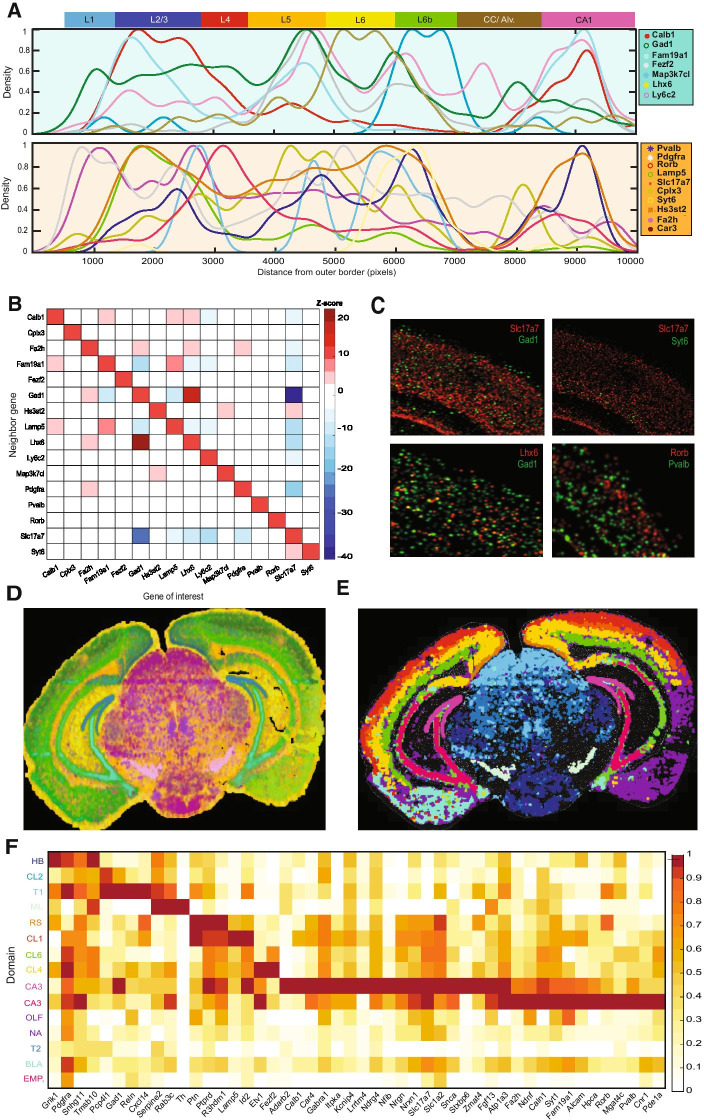
Analysis of the expression of 17 genes in the mouse cortex. **A** One dimensional KDE estimation of the expression of the 17 genes along the dorso-ventral axis of the cortex. Genes are randomly divided in two line plots to facilitate their comprehension. **B** Heat map representing the colocalization between the genes analyzed. Positive Z-scores (red) represent colocalization of the genes and negative Z-score (blue) represent mutually exclusive expression. **C** KDE of the expression of several different genes, represented pairwise. Different co-expression patterns are represented including mutually exclusive genes (top,left),colocalizing genes (down,left), partially colocalizing genes (top,right) and genes with non-related expression patterns (down,right). **D** Two-dimensional map of the bins generated when segmenting the mouse coronal section. Color code corresponds to the RGB loadings of each bin’s score on the top three UMAP components found when doing dimensionality reduction analysis. Different colors, indicating different loadings for each of components are found in different areas of the brain, highlighting the difference in expression found for the genes included in the panel. **E** Two-dimensional map of the bins generated previously, where each color represents one of the 15 clusters defined by performing hierarchical clustering on the segmented dataset. **F** Mean expression of each of the clusters defined in E for all the genes included in the analysis. The colors of each cluster, on the Y axis, correspond to the colors used in **E** for each cluster

In order to demonstrate the capacity of *Matisse*, we reanalyzed the published HybISS dataset of a mouse coronal section [15]. A subset of 17 genes, out of the 119 present in the original data, were selected for a comprehensive, read-based analysis, of their spatial expression in the primary visual cortex (Visp). A main outer-inner gradient was found de novo by analyzing the KDE expression profiles of these genes together [see Additional file 1], in agreement with the well-known layer-based structure of the whole mouse cortex [28] and, in particular, within the Visp. This one-dimensional study of the gradient reveals gene expression changes through the different cortical layers, with different profiles, from layer-specific markers such as *Rorb* to general excitatory markers spanning all layers such as *Slc17a7* (Fig. 2A). Gene colocalization can also been explored, showing for example correlations between inhibitory markers, such as *Gad1* and *Lhx6*, and mutually exclusive expression patterns for excitatory and inhibitory markers, like *Slc17a7 and Gad1, respectively* (Fig. 2B, C).

### Segmented datasets

*Matisse* includes different segmentation strategies that can be applied to ISS expression maps depending on the question addressed and the quality of the data. These strategies have been evaluated using the gene expression maps for all the 119 genes in the HybISS dataset. To find region-specific expression patterns within tissue sections, adjustable bins can be used for segmentation. Here, equally distributed bins with a radius of 250 pixels and 50 pixels between them were created. Bins outside a range of expression were excluded from the analysis as well as low expressed genes that could bias the results [see Additional file 2, 3]. The RGB representation of the 3 first UMAP dimensions of the dataset revealed main expression differences between the cortex and the hippocampus compared to the thalamus (Fig. 2C), as well as a gradual change of expression in the outer-inner axis of the cortex. To further study the main expression differences shown in different regions of the tissue, PCA was performed, here identifying additional expression-unique regions [see Additional file 2, 3]. Hierarchical clustering can be performed on the binned data to spatially define de novo expression specific regions. As exemplified by using the 119 gene dataset, this resulted in 15 different expression areas (Fig. 2E). Main anatomical regions of the brain could be differentiated by their expression, including different specific regions within the thalamus; CA1-3 in the hippocampus and different layers within the cortex. Most of the regions defined presented specific markers within the panel (Fig. 2F). Regions defined were found conserved between the two hemispheres of the section in most cases, suggesting the relevance of the regions defined.

#### Integration of single-cell RNA-sequencing data

**Fig. 3:**
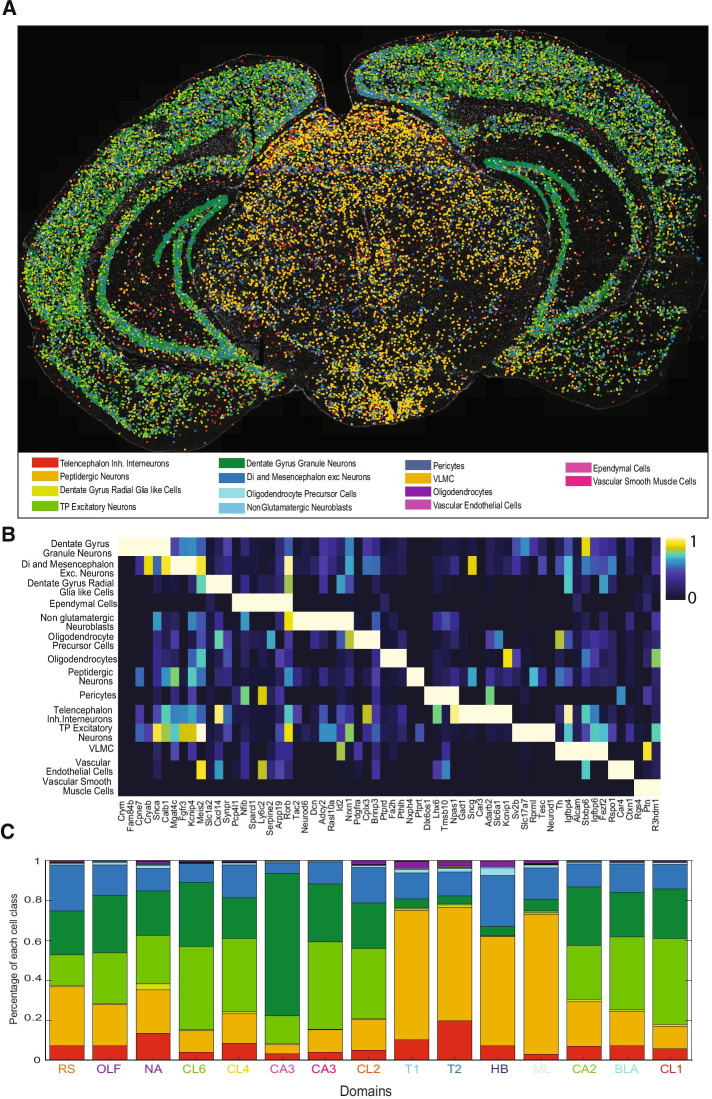
Probabilistic cell typing of individual cells in a mouse coronal section. **A** Map of the most probable cell type predicted for each cell segmented in the mouse coronal section analyzed. Cells have been classified in 14 different major classes defined by *Zeisel *et al*.* [29] based on the expression levels of all the genes analyzed in the ISS experiment. **B** Heat map representing the mean expression of each gene for the cells assigned to each specific class by probabilistic cell typing (pciSeq) normalized the total expression of each gene. Differentially enriched genes are found for each of the cell classed identified. **C** Relative distribution of the classes assigned on **A** for each of the domains defined in Fig. 2E. Domain colors correspond to the colors used in Fig. 2E to visualize the domain on each class and cell class colors correspond to the ones used in **A** to represent the most probable cell class assigned to each cell

Segmentation of individual cells was performed by DAPI nuclear staining in order to explore the cellular diversity. Leveraging scRNA-seq data, pciSeq [20] was applied to the dataset in order to spatially map the main cell classes present in the sample, based on the expression patterns of the classes described in Zeisel et al. [29]*.* 14 different classes were found in the section analyzed (Fig. 3A). Different relative abundances of the defined cell types were found among the spatial domains described in Fig. 2E, varying from close to domain-specific classes, like Vascular and Leptomeningeal cells, with a high frequency in domain 29 only; to widely spread classes, like telencephalon and mesencephalon excitatory neurons. The inclusion of these functionalities, and coupling them in *Matisse* allows the user not only to place the cell types identified by scRNA-seq in a spatial context, but also to identify supra-cellular spatial domains with a specific cellular composition.

#### Integration of datasets from other spatial technologies

**Fig. 4:**
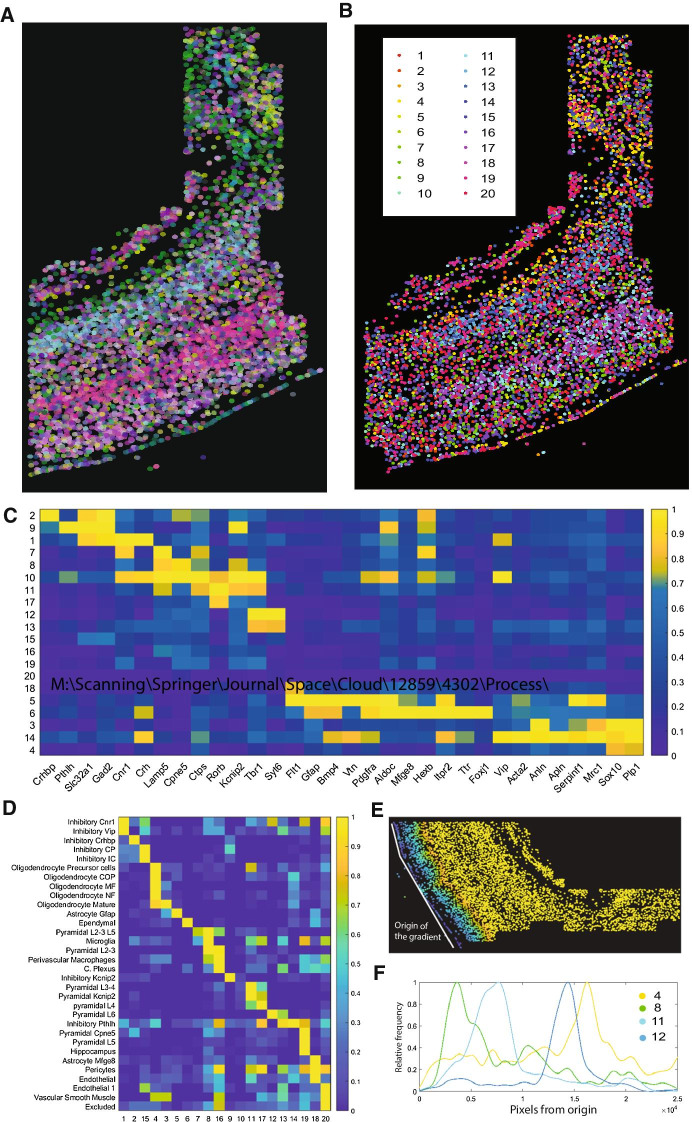
Integration of different spatial technologies to undercover biological patterns using *Matisse*. **A** Spatial map of the osmFISH dataset, where every cell is colored by an RGB representation of 3 first dimensions of a UMAP representation. **B** Spatial map of the osmFISH dataset, where every cell has been assigned to a cluster found by hierarchical clustering. **C** Mean expression of every gene in the 20 clusters found de novo in the osmFISH dataset. **D** Correspondance between the predicted de novo clusters and cell types assigned to each cell in Codeluppi et al. [6]. **E** Gradient defined in the osmFISH dataset, where the white line represents the origin of the gradient and cells are colored depending on the minimum distance from itself to the origin line. Blue colors represent smaller distances, whereas yellow colors represent bigger distances. **F** Kernel density estimation of the frequency of 4 specific clusters (4,8,11 and 13) along the one-dimensional axis defined in **E**

To prove that Matisse is able to integrate samples from different image-based spatially resolved transcriptomic technologies*,* we used cell maps created using osmFISH, described in Codeluppi et al*.* [6] We performed dimensionality reduction on the osmFISH dataset, representing each spot with the composite color obtained from transforming their 3 dimensional UMAP scores to RGB (Fig. 4A). We then performed hierarchical clustering, identifying a set of 20 clusters (Fig. 4B). Each cluster found exhibited a specific expression signature and corresponded to specific cell types identified by Codeluppi et al. [6] (Fig. 4C, D). We also used *Matisse* to represent the location of some of the clusters along the dorso-ventral axis of the cortex, identifying different spatial patterns for the different clusters along this axis (Fig. 4E, F).

## Discussion

Here we describe *Matisse*, a Matlab toolbox specifically designed for a comprehensive initial analysis of ISS datasets. As an example, we have used its main analytical tools to better describe the 2-dimensional expression pattern of 119 genes in the whole mouse coronal section from Gyllborg et al*.* [30]. In addition, we have proved the capacity of *Matisse* to integrate other spatial transcriptomic datasets by analyzing datasets from osmFISH.

*Matisse* summarizes in a toolbox the most important analytical methods applied so far to 2-dimensional ISS datasets, and adds to these some new functionalities such as colocalization, co-expression, dimensional reduction, feature selection and quality control tools, that can help in the interpretation of spatial datasets. In contrast with the rest of the available packages, *Matisse* can be used to analyze both segmented and unsegmented datasets. This can be extremely useful for tissues where cell segmentation cannot be achieved, or in sparse datasets, where the number of reads/cell obtained is low, and the study of individual cells can be driven by stochastic effects. In the case of functions for segmentation failing to segment individual cells, such as in cell dense rich areas, they can be used to identify spatial domains and molecular signatures present in the datasets analyzed.

The toolbox also includes some of the most common clustering approaches used for the analysis of single-cell datasets, although it lacks other widely used algorithms, such as Leiden or Louvain [31] since they lack an implementation in MATLAB. However, the different algorithms included are suitable for an initial exploration of the data. In addition, we added pciSeq as a supervised cell typing algorithm, which uses the expression of cell types characterized via scRNA-seq to classify the cells within a section.

An interesting capacity included in *Matisse* is the possibility to process several samples at the same time. Since ISS can be used with low magnification objectives, one can interrogate multiple samples in the same experiment in a cost and time-efficient manner. Therefore, tools designed to process ISS datasets should be able to integrate multiple samples in the same analysis. As with other -omic approaches, technical variation is expected to occur between samples, due to the origin of the samples, their RNA quality, or differences in their processing. In this case, harmonization would be required to remove the technical variability to resolve cross sample similarities and differences. Due to the lack of statistical tools specifically designed for spatially-resolved transcriptomics datasets, methods originally designed for other -omics technologies have been adopted by the field, especially for comparing spatial-based and scRNA-seq datsets [32]. Within *Matisse*, we have implemented *Combat* [33], which is a known method designed for batch effect correction of RNA sequencing datasets and that, therefore, should be sufficient to correct the batch effects between datasets produced by the same technology with similar detection efficiencies of each gene but varying RNA quality. However, using algorithms designed for other -omic technologies within spatially resolved approaches misses on a key aspect which is the spatial distribution of individual transcripts. It is therefore vital for the development of new analytical tools that are specific for image-based spatial technologies to compare multiple sample datasets.

Despite the increasing amount of analytical tools designed for the exploration of spatially resolved transcriptomics datasets, most of them have been created with the aim of performing specific analysis in a precise manner [10], usually compromising the time and the versatility of it. Since the number of laboratories using spatially-resolved methods are increasing rapidly, we believe that apart from specific tools, there is a need for fast, versatile, comprehensive and easy to use tools. Therefore, the tools implemented in the toolbox presented can be tuned to either run fast and preliminary or for more precise analysis, intending to help the user to obtain the biologically relevant information efficiently.

*Matisse* has been designed to consider the specific requirements of ISS analysis, such as handling large sample areas, processing multiple datasets and dealing with gene panels containing from 50 to 200 genes, its application in the analysis of other kind of datasets is also possible, with the only condition of having as an input the position of the reads decoded, or the position and expression of individual cells/bins. Despite being generated with different chemistries, the nature of all image-based spatially resolved transcriptomics methods, resolving individual spots in a spatial context, facilitates their convergence on the analysis side. In this sense, the community is moving towards the development of tools able to integrate datasets from different sources, both in the preprocessing [34] and downstream analysis side [25]. This is essential to maximize the variety of analytical tools available to analyze these datasets and to minimize the development of already existent tools.

## Conclusions

*Matisse* provides a user-friendly and versatile toolbox to explore ISS datasets which can help new users to understand the main biological information captured in an ISS experiment. This tool allows the analysis of both unsegmented and segmented datasets, exploring different levels of complexity within a dataset in a consistent manner*.* In addition, several datasets can be integrated into the same analysis in a comprehensive manner, opening the possibility, thanks to harmonization strategies, of combining spatially resolved transcriptomics technologies into the same analysis.

## Availability and requirements

- **Project name:** Matisse.
- **Project home page:** https://github.com/Moldia/Matisse
- **Operating system(s):** Windows, Linux, Mac OS.
- **Programming language:** MATLAB. Implemented in MATLAB 2019b.
- **Other requirements: MATISSE requires different** MATLAB add-ons, including the Bioinformatics and Image Processing Toolbox.
- **License:** GNU.
- **Any restrictions to use by non-academics:** No.

## Supplementary Information

Additional file 1: Coronal brain region selected for analysis and KDE plots. A. Regions of interest (ROI) analyzed in Figure 2A (blue) and Figure 2.B-C/Additional file 1.D (yellow) displayed over the DAPI staining from the mouse coronal section explored in Gyllborg et al^30^. B. Regional localization of mouse brain coronal section, indicating the approximate location of the regions of interest (ROI) analyzed in Figure 2A (blue) and Figure 2B-C/Additional file 1.D (yellow). Image credit: Allen Brain Institute. C. Main gradient found de novo in the mouse coronal cortex ROI (blue square in Additional file 1A), which indicates the different gene expression found between the different layers of the mouse cortex. D. KDE plots of 14 of the genes studied in detail in yellow ROI defined in Additional File 1. A grayscale color map is used to represent the level of expression of the different genes, where white represents high expression while black represents lack of expression.

Additional file 2: Quality control and principal component analysis of bins in the mouse section. A. Distribution of the number of reads found on each bin. Dashed red line indicates the minimum number of reads/cell required and dashed green line indicates the maximum number of reads accepted. B. Distribution of the number reads found for each gene in the sample analyzed. The dashed red line indicates the minimum number of reads required for a gene to be included in further analysis. C. Percentage of variable explained by each principal component found when performing PCA on the bins accomplishing the QC requirements from Additional file 2A and Additional file 2B. D. Score of each of the bins for the top 10 principal components found in the binned dataset. Red indicates high score in a specific bin and blue indicates low score. Differentially expressed regions are found when exploring each of the 10 principal components.

Additional file 3: Correlation between the expression of genes and principal components. A. Heat map representing the correlation between the expression of every gene and the top 10 principal components’ scores bins described in Figure 2D-E. Low correlations are labeled in blue while high correlations are shown in white, as shown in the color bar found in the right of the heat map.

## Data Availability

An stable version of *Matisse*, the analysis toolbox described in this manuscript, can be downloaded from https://www.mathworks.com/matlabcentral/fileexchange/91195-matisse_rev, where the documentation can be also found. The most updated version of the toolbox can also be downloaded from https://github.com/Moldia/Matisse. The datasets analyzed during the current study are available in at Figshare (https://doi.org/10.17045/sthlmuni.12821840), in the case of the HybISS [19] datasets. In the case of the osmFISH [6] datasets, the datasets analyzed during the current study can be found online at http://linnarssonlab.org/osmFISH/.

## Abbreviations

HybISS: Hybridization-based in situ sequencing
ISS: In situ sequencing
KDE: Kernel density estimation
Matisse: MATLAB analysis toolbox for In Situ Sequencing Expression maps
scRNA-seq: Single Cell RNA sequencing
osmFISH: Ouroboros single molecule FISH
PCA: Principal component analysis
pciSeq: Probabilistic cell typing by in situ sequencing
tSNE: T-distributed stochastic neighbor embedding
UMAP: Uniform Manifold Approximation and Projection
