# Supplementary figures and images for: Matisse: a MATLAB-based analysis toolbox for in situ sequencing expression maps
Source: BMC Bioinformatics. 2021 Jul 31;22:391. doi: 10.1186/s12859-021-04302-5 (PMC8325818; doi:10.1186/s12859-021-04302-5)

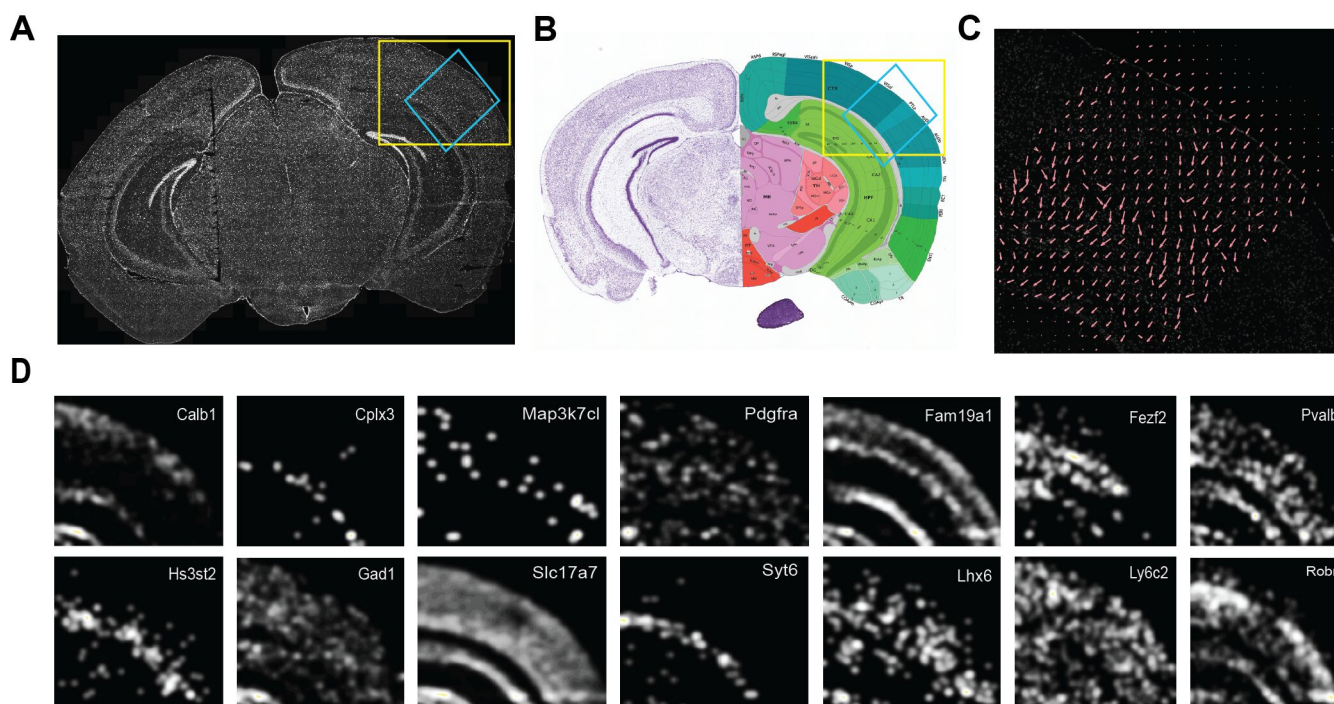

Supplement: Supplementary file 1 — Coronal brain region selected for analysis and KDE plots. A. Regions of interest (ROI) analyzed in Figure 2A (blue) and Figure 2.B-C/Additional file 1.D (yellow) displayed over the DAPI staining from the mouse coronal section explored in Gyllborg et al30. B. Regional localization of mouse brain coronal section, indicating the approximate location of the regions of interest (ROI) analyzed in Figure 2A (blue) and Figure 2B-C/Additional file 1.D (yellow). Image credit: Allen Brain Institute. C. Main gradient found de novo in the mouse coronal cortex ROI (blue square in Additional file 1A), which indicates the different gene expression found between the different layers of the mouse cortex. D. KDE plots of 14 of the genes studied in detail in yellow ROI defined in Additional File 1. A grayscale color map is used to represent the level of expression of the different genes, where white represents high expression while black represents lack of expression. [file 12859_2021_4302_MOESM1_ESM.pdf]

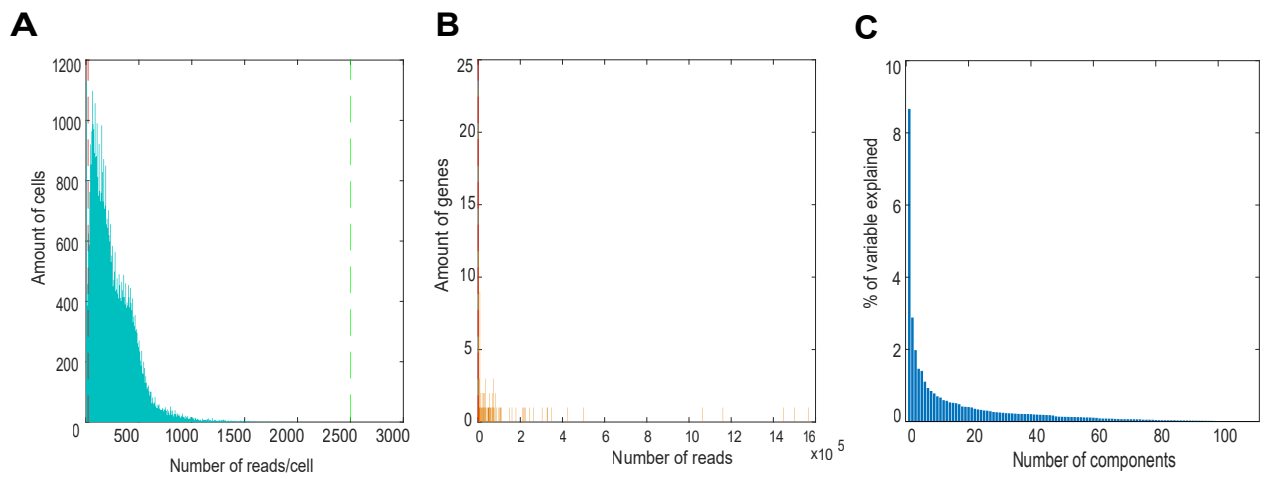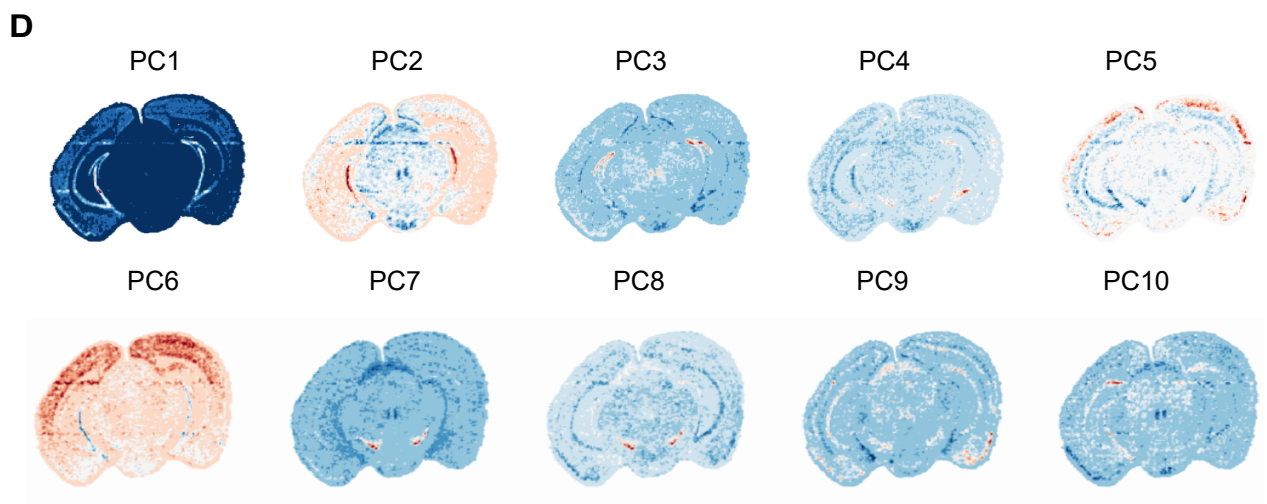

Supplement: Supplementary file 2 — Quality control and principal component analysis of bins in the mouse section. A. Distribution of the number of reads found on each bin. Dashed red line indicates the minimum number of reads/cell required and dashed green line indicates the maximum number of reads accepted. B. Distribution of the number reads found for each gene in the sample analyzed. The dashed red line indicates the minimum number of reads required for a gene to be included in further analysis. C. Percentage of variable explained by each principal component found when performing PCA on the bins accomplishing the QC requirements from Additional file 2A and Additional file 2B. D. Score of each of the bins for the top 10 principal components found in the binned dataset. Red indicates high score in a specific bin and blue indicates low score. Differentially expressed regions are found when exploring each of the 10 principal components. [file 12859_2021_4302_MOESM2_ESM.pdf]

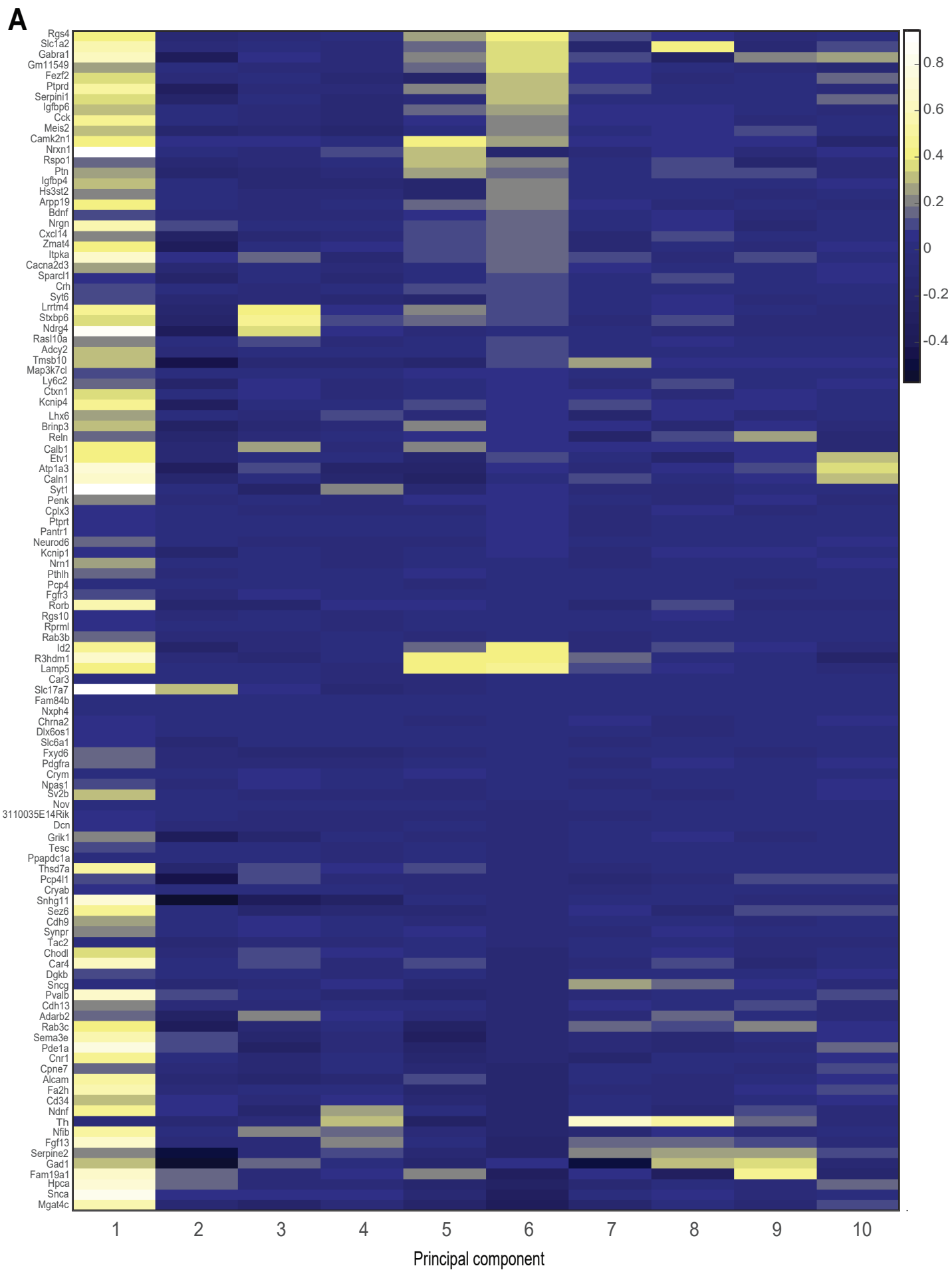

Supplement: Supplementary file 3 — Correlation between the expression of genes and principal components. A. Heat map representing the correlation between the expression of every gene and the top 10 principal components’ scores bins described in Figure 2D-E. Low correlations are labeled in blue while high correlations are shown in white, as shown in the color bar found in the right of the heat map. [file 12859_2021_4302_MOESM3_ESM.pdf]
